# Gene cloning and expression of MAP30 in *Pichia pastoris*

**DOI:** 10.1080/13102818.2014.901667

**Published:** 2014-06-04

**Authors:** Fang Wang, Chun-yu Chi, Li-yuan Wang, Yu Qiao, Xiao-xia Jin, Guo-hua Ding

**Affiliations:** ^a^ Heilongjiang Provincial Key Laboratory of Plant Biology, Life Science and Technology College, Harbin Normal University, Harbin, China

**Keywords:** expression, pGAPHα, MAP30, *Pichia pastoris*, clone

## Abstract

MAP30, a single-stranded type-I ribosome inactivating protein found in *Momordica charantia*, shows anti-HIV and anti-tumour activity. It could significantly inhibit the HIV-1 and herpes simplex virus infection. In this study, we tried a safe and convenient expression system supplying MAP30 protein for medical practice. The gene encoding MAP30 was cloned into pMD18-T vector. The pMD18-*MAP*30 plasmid was transformed into competent *Escherichia coli* JM109 by a chemical method. The *MAP*30 gene was obtained from the pMD18-*MAP*30 plasmid digested with *Not*I and *SnaB*I and the *MAP*30 gene was ligated into pGAPHα. Then, pGAPHα-*MAP*30 was transformed into *Pichia pastoris* GS115 by electroporation. GS115 transformants were analysed by sodium dodecyl sulfate polyacrylamide gelelectrophoresis (SDS-PAGE) and Western blot. SDS-PAGE revealed an extra band of approximately 32 kDa in the supernatant protein of the GS115 transformants and in their intracellular protein fraction. The result of Western-blot analysis showed that the supernatant and the cell pellet from GS115 with pGAPHα-*MAP*30 could specially bind to monoclonal antibodies against His in the 32 kDa site. These results demonstrated that the expression of MAP30 in *P. pastoris* was successful; the process of the expression did not need methanol induction or introduction of an antibiotic-resistance gene. The study may provide a new way for MAP30 synthesis. Owing to its safety, this new approach is expected to be widely used in the medical field.

## Introduction

MAP30 was first purified by Lee Huang from seeds and fruits of *Momordica charantia* in 1990.[1] It is a single-stranded, type-I ribosome inactivating protein containing 263 amino acids. The full length gene encoding MAP30 is 861 bp and has no introns. MAP30 has been reported to possess anti-HIV and anti-tumour activity, which could significantly inhibit the HIV-1 and herpes simplex virus infection.[2,3] Furthermore, MAP30 inhibited the proliferation of AIDS-related lymphoma cells infected with Kaposi's sarcoma-associated virus by modulation of different viral and cellular genes.[4] At the same time, it could selectively attack tumour-transformed and HIV-infected cells, and has no adverse effects on normal cells. MAP30 has a significant application value in clinical studies.

Recombinant MAP30 could be expressed in different systems. For example, it was expressed in an *Escherichia coli* expression system,[5] which exhibited fast and robust growth in bioreactors using simple media. However, the expression system had some disadvantages that it cannot perform adequate post-translational processing of many polypeptides and the products are insoluble or incorrectly folded.[6] In recent years, with the development of biotechnology, the *Pichia pastoris* expression system is widely used in producing recombinant proteins. *P. pastoris* is as easy to manipulate as *E. coli* and has some additional advantages of higher eukaryotic expression systems, e.g. protein processing, protein folding and post-translational modification.[7–9] The *P. pastoris* expression system is faster, easier, and less expensive to use than other eukaryotic expression systems, and generally gives a higher expression level.[10] The expression vectors for *P. pastoris* are quite different, such as pPIC9K and pGAPZα, but they could not be used in the food industry because of the need of methanol in expression or the introduction of an antibiotic-resistance gene by transformation. A neotype secreting expression vector for *P. pastoris* (pGAPHα) was constructed by Northeast Agricultural University, Harbin, China. The methanol-induced Alcohol oxidase (AOX) promoter of the vector was replaced by a Glyceraldehyde-3-phosphate dehydrogenase (GAP) promoter, and made *P. pastoris* expression not dependent on methanol induction or antibiotic-resistance gene.[11]

In the present study, to develop an efficient and safe expression of MAP30, the pGAPHα expression vector was used to produce MAP30 in a *P. pastoris* expression system. The study will lay the foundation for further developments for the needs of the medical field.

## Materials and methods

### Chemicals and reagents

All the restriction enzymes, T_4_DNA ligase and Taq DNA polymerase were from TaKaRa Biotechnology (Dalian, China). Plasmid pGAPHα and *P. pastoris* GS115 were obtained from Northeast Agricultural University. Primers were synthesised by Sangon Biotech (Shanghai, China).

### DNA preparation and cloning of the *MAP30* gene

The genomic DNA of *M. charantia* was obtained by the Cetyltrimethyl ammonium bromide (CTAB) method from fruits of *M. charantia*. The three different primers were designed according to the *MAP*30 gene sequence in GenBank (s79450). As the sequence of the reverse primer was longer, two primers were designed for the reverse primer:

- F1(5′TACGTAATCTTCATTGGTGTTCCTGCTGCCAA 3′)
- R1(5′ATGATGATGATGATGATGATTCACAACAGATTCCCC3′)
- R2(5′GCGGCCGCTCAATGATGATGATGATG3′

The full length sequence of the *MAP*30 gene was Polymerase chain reaction (PCR) amplified from the genomic DNA of *M. charantia*, using F1/R1 and F1/R2. Amplification conditions were: preheating at 94 °C for 1 min; 30 cycles at 94 °C for 30 s, 56 °C for 30 s, and 72 °C for 45 s; followed by final extension at 72 °C for 5 min. An 860 bp fragment was recovered from the gel by the UNIQ-10 Gel Extraction Kit (Sangon Biotech) and cloned into a pMD18-T vector. The pMD18-*MAP*30 plasmid was transformed into the competent *E. coli* JM109 by the CaCl_2_ method. The nucleotide sequence of *MAP*30 gene was confirmed by sequencing. The pMD18-*MAP*30 plasmid was digested with *Not*I and *Sna*BI, and the 860 bp DNA fragment was recovered from the gel by the UNIQ-10 Gel Extraction Kit, and then ligated to the *P. pastoris* expression vector pGAPHα digested with restriction enzymes *Not*I and *Sna*BI. The constructed vector, pGAPHα-*MAP*30 was transformed into competent *E. coli* DH5α. The *MAP*30 gene of the plasmid was confirmed by restriction enzyme analysis and by sequencing.

### Transformation of *P. pastoris* and screening of transformants

*P. pastoris* GS115 strain was mixed with *Bgl*II-linearised pGAPHα-*MAP*30 and electroporated in a 0.2-cm electroporation cuvette using a Bio Rad Gene Pulser. Immediately after the pulsing, 1 mL of cold 1 mol/L sorbitol was added to the *P. pastoris* cells and plated on MD. His^+^ transformants were selected on MD plates (13.4 g/L YNB, 0.4 mg/L biotin, 20 g/L dextrose, 20 g/L agar) and incubated at 28 °C. The integration of the *MAP*30 gene coding sequence in the genome of *P. pastoris* was confirmed by Colony PCR using single colonies from MD plates as templates and specific primers (F1, R2).

### Expression of recombinant MAP30 in GS115 strain and Western blotting

A single colony of GS115 transformant was grown in 50 mL of YPD (1% Yeast extract, 2% peptone, 2% dextrose) at 28 °C in a shaking incubator (200 r/min) to OD_600_ = 4. The cell culture was centrifuged; then the cell pellet and the supernatant from YPD expression culture medium were harvested separately. The intracellular protein of the GS115 transformant cell pellet and the concentrated supernatant of the GS115 transformant culture media were withdrawn for assaying for intracellular expression and secreted expression by SDS-PAGE and Western blot using rabbit anti-His antibody. The concentrated supernatant protein of GS115 culture media was used as a negative control.

## Results and discussion

Given the anti-HIV and anti-tumour activity of MAP30, it has high medicinal value. In recent years, several attempts to express MAP30 expression have been reported. All kinds of expression systems were applied to MAP30 expression, but these expression systems were not safe and were not applicable to medical practice. In our study, MAP30 protein was successfully expressed in *P. pastoris* GS115 by using a pGAPHα expression vector.

### Cloning of the *MAP*30 gene

**Figure 1.**
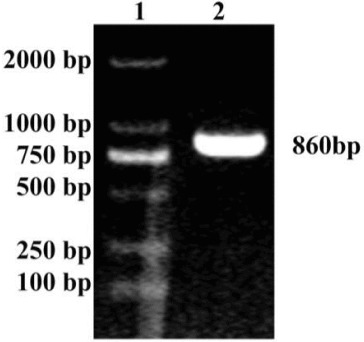
PCR product of the *MAP*30 gene. Lane 1: DL 2000 marker; Lane 2: PCR product of the *MAP*30 gene.

The product amplified by primers F1 and R2 revealed a band of approximately 860 bp in the agarose gel (Figure 1). The band size was similar to the expected band for *MAP*30. The *MAP*30 gene was cloned into the pMD18-T vector to generate a recombinant pMD18-*MAP*30 plasmid. The recombinant was confirmed by sequencing. Then the pMD18-*MAP*30 plasmid was digested with *Not*I and *Sna*BI, the 860 bp digestion of pMD18-*MAP*30 was ligated into pGAPHα to generate a recombinant pGAPHα-*MAP*30. The digestion of pGAPHα-*MAP*30 with *Not*I and *Sna*BI resulted in two fragments sized 8100 and 860 bp, indicating that the clone was right. The *MAP*30 gene of pGAPHα-*MAP*30 was also confirmed by sequencing; the sequencing result was compared to the National Center for biotechnology information (NCBI) GenBank by basic local alignment search tool (BLAST).

### Transformation of *P. pastoris* and selection of transformants

The transformants with multiple copy inserts and His^+^ phenotype were screened out in MD, Histidine deficient medium. The integration of *MAP*30 gene in the genome of *P. pastoris* was confirmed by Colony PCR.

### SDS-PAGE analysis of *MAP*30 in *P. pastoris* GS115 and Western blot

**Figure 2.**
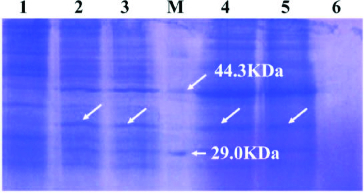
SDS-PAGE analysis of GS115 transformant. Lane 1: intracellular protein of GS115 pellet; Lanes 2 and 3: intracellular protein of GS115 transformant; Lane M: protein marker; Lanes 4 and 5: supernatant protein of GS115 transformant; Lane 6: supernatant protein of GS115.

**Figure 3.**
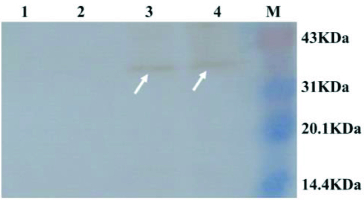
Western blot analysis of GS115 transformant. Lane 1: supernatant of GS115; Lane 2: cell pellet of GS115; Lane 3: supernatant of GS115 tranformant with pGAPHα-*MAP*30; Lane 4: cell pellet of GS115 transformant with pGAPHα-*MAP*30; Lane M: pre-stained protein molecular weight marker.

The supernatant of GS115 transformant with pGAPHα-*MAP*30 and its cell pellet were collected separately. The intracellular protein and the concentrated supernatant protein were detected by SDS-PAGE. Compared with the negative control, an extra band of approximately 32 kDa was found in the supernatant protein and the intracellular protein fraction (Figure 2). The protein size of approximately 32 kDa was nearly identical to the protein size of MAP30.[12] The Western blot result showed that the supernatant and the cell pellet from GS115 with pGAPHα-*MAP*30 could specially bind to monoclonal antibodies against His in the 32 kDa site, but the supernatant and the cell pellet from GS115 could not (Figure 3). The result revealed that the expression of MAP30 in *P. pastoris* was successful.

Compared with the *E. coli* expression system, MAP30 protein could be folded properly and glycosylated in the *P. pastoris* expression system.[13] What is more, compared with the pPIC9K and pGAPZα expression vector, the expression of MAP30 using the pGAPHα expression vector does not need methanol induction or introduction of an antibiotic-resistance gene. In the expression system, MAP30 protein could be secreted directly to the supernatant by α-factor. The approach proposed in our study makes MAP30 expression more safe and is expected to be widely used in the medical field. However, the yield of MAP30 protein was lower. Therefore, further improvement is needed.

## Conclusions

In the present study, MAP30 protein was successfully expressed in *P. pastoris* GS115 by using a pGAPHα expression vector. Secreted and intracellular MAP30 protein was obtained from GS115 transformants with pGAPHα-*MAP*30. The expression vector used does not require methanol induction or introduction of an antibiotic-resistant gene, which makes MAP30 expression safer. In the proposed expression system, MAP30 protein could be secreted directly to the supernatant by α-factor, and the transformants with multiple copy inserts and His^+^ phenotype were screened out in MD, Histidine-deficient medium. The proposed approach is expected to be widely used in the medical field but the achieved yield of MAP30 needs to be further improved.
